# Hyaluronic Acid Coated Chitosan Nanoparticles Reduced the Immunogenicity of the Formed Protein Corona

**DOI:** 10.1038/s41598-017-10836-7

**Published:** 2017-09-05

**Authors:** Abdulaziz Almalik, Hicham Benabdelkamel, Afshan Masood, Ibrahim O. Alanazi, Ibrahim Alradwan, Majed A. Majrashi, Assim A. Alfadda, Waleed M. Alghamdi, Haitham Alrabiah, Nicola Tirelli, Ali H. Alhasan

**Affiliations:** 1 National Center for Biotechnology, Life science and Environment Research Institute, King Abdulaziz City for Science and Technology (KACST), P.O. Box 6086, Riyadh, 11461 Saudi Arabia; 2 Proteomics Resource Unit, Obesity Research Center, College of Medicine, King Saud University, P.O. Box 2925, Riyadh, 11461 Saudi Arabia; 3 The National Center for Genomic Technology (NCGT), Life science and Environment Research Institute, King Abdulaziz City for Science and Technology (KACST), P.O. Box 6086, Riyadh, 11461 Saudi Arabia; 4 Department of Pharmaceutical Chemistry, College of Pharmacy, King Saud University, P.O. Box 2457, Riyadh, 11451 Saudi Arabia; 5 NorthWest Centre for Advanced Drug Delivery (NoWCADD), Division of Pharmacy and Optometry, School of Health Sciences, University of Manchester, Manchester, UK

## Abstract

Studying the interactions of nanoparticles (NPs) with serum proteins is necessary for the rational development of nanocarriers. Optimum surface chemistry is a key consideration to modulate the formation of the serum protein corona (PC) and the resultant immune response. We investigated the constituent of the PC formed by hyaluronic acid-coated chitosan NPs (HA-CS NPs). Non-decorated chitosan NPs (CS NPs) and alginate-coated chitosan NPs (Alg-CS NPs) were utilized as controls. Results show that HA surface modifications significantly reduced protein adsorption relative to controls. Gene Ontology analysis demonstrates that HA-CS NPs were the least immunogenic nanocarriers. Indeed, less inflammatory proteins were adsorbed onto HA-CS NPs as opposed to CS and Alg-CS NPs. Interestingly, HA-CS NPs differentially adsorbed two unique anti-inflammatory proteins (ITIH4 and AGP), which were absent from the PC of both controls. On the other hand, CS and Alg-CS NPs selectively adsorbed a proinflammatory protein (Clusterin) that was not found on the surfaces of HA-CS NPs. While further studies are needed to investigate abilities of the PCs of only ITIH4 and AGP to modulate the interaction of NPs with the host immune system, our results suggest that this proof-of-concept could potentially be utilized to reduce the immunogenicity of a wide range of nanomaterials.

## Introduction

The application of nanotechnology in medicine (i.e. nanomedicine) has the potential to considerably improve medical practices both in the treatment and in the diagnosis of diseases^1^. Here, we focus on drug delivery, where nanomaterials can impact specificity, safety, and efficacy of an active drug^1, 2^. Since they are used in contact or within the human body, and often exposed to blood, the nanomaterials have to satisfy criteria of bio/hemocompatibility. However, this evaluation is complicated by the surface dynamics of the nanomaterials once exposed to a biological environment: their interactions with biomolecules often lead to the formation of a “protein corona (PC)”^3, 4^. PC is a layer of adsorbed biomolecules, which at the same time minimizes the surface energy of the nanomaterial, and changes its surface to the point that it bears little resemblance to the original nanomaterial in terms of chemical composition, physico-chemical interactions and biological responses it can elicit^5^. As an example of the latter effect, nanoparticles (NPs) that absorb opsonins with high affinity tend to be rapidly cleared from blood stream^6^. Therefore, studying the composition of a PC formed around a nanomaterial could allow for the rational functionalization of NPs with anti-inflammatory proteins to prolong their blood circulation times and bioavailability.

Nanomaterials prepared from polysaccharides have been extensively exploited for drug delivery, because of their natural origin, chemical functionality, biodegradability, and good biocompatibility. Here we investigated chitosan (CS), which is a copolymer of β-(1 → 4)-linked D-glucose-2-amine and N-acetyl-D-glucose-2-amine. CS is extensively used in a broad spectrum of applications ranging from food manufacturing to tissue engineering and delivery of macromolecular payloads (e.g. nucleic acids, proteins)^7^. When CS is the main component of the NPs (CS NPs), these systems possess a cationic surface, which significantly reduces their circulation time and bioavailability upon exposure to a biological environment^8, 9^. However, when these NPs are decorated with anionic polysaccharides, such as hyaluronic acid (HA) or alginate (Alg), both protein adsorption and the rate of macrophage uptake decreased^10, 11^. These effects have been ascribed, at least in part, to the rapid and unselective interactions of cationic NPs with serum proteins that trigger phagocytosis. Importantly, the presence of HA on the surface of CS NPs (HA-CS NPs) allows for targeted delivery to cells bearing CD44 receptors as opposed to nonspecific cell-uptake mechanisms of the plain CS NPs^12–14^.

In this study, we investigated the effect of coating CS NPs with HA on the compositions of the formed PCs. In short, we profiled the protein identities of the formed corona on HA-CS NPs. Non-decorated chitosan NPs (CS NPs) and alginate-coated chitosan NPs (Alg-CS NPs) were used as controls. Specifically, we report a proteomics analysis of bovine serum proteins associated to the three kinds of particles, using proteome-mapping techniques followed by Gene Ontology analysis.

## Results and Discussion

**Figure 1:**
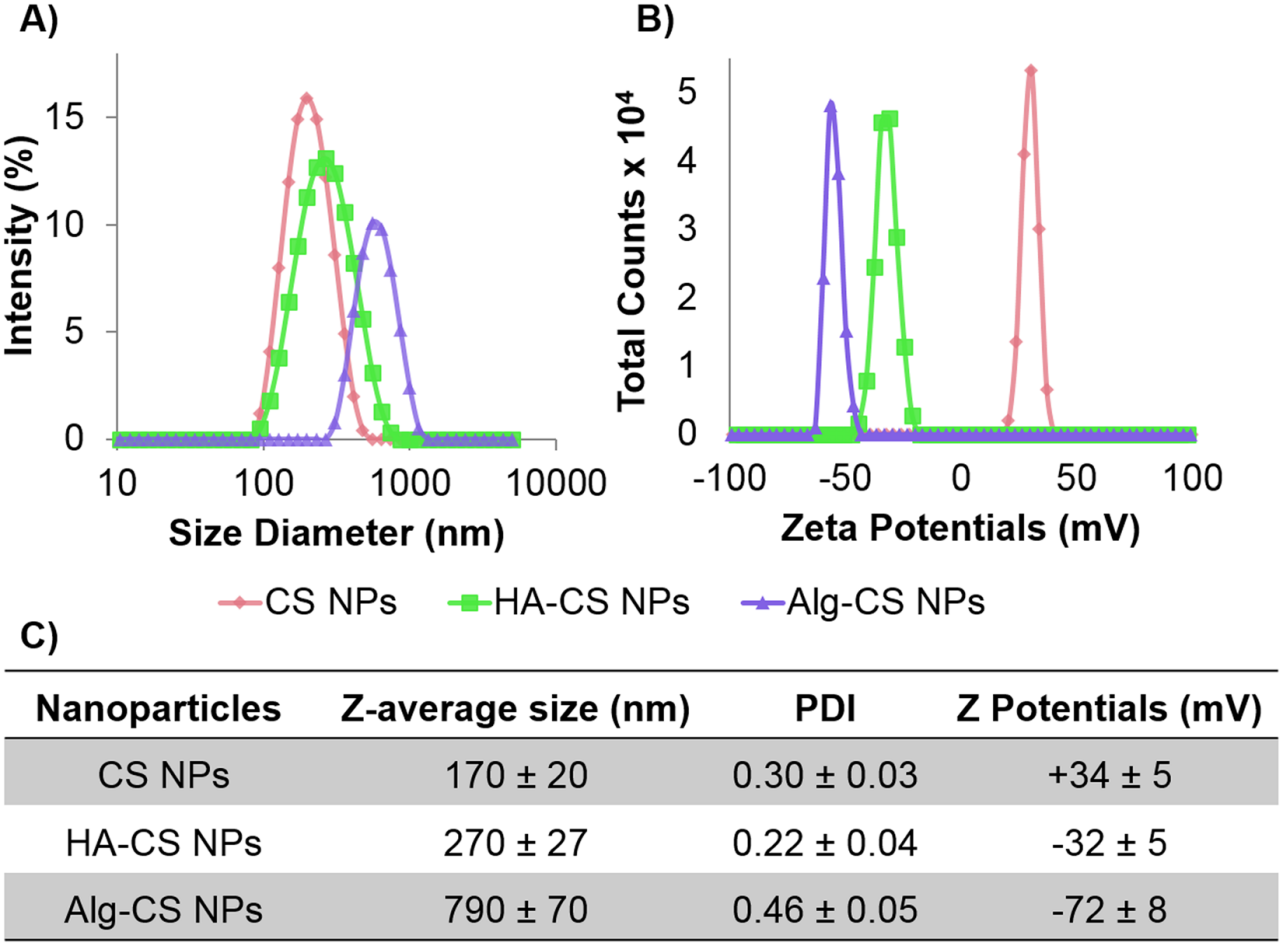
Size and surface chemistry characterization of nanoparticles. (**A**) Size and (**B**) zeta potential distributions of chitosan nanoparticles (CS NPs), hyaluronic acid-coated CS NPs (HA-CS NPs), and alginate-coated CS NPs (Alg-CS NPs). (**C**) Statistical analysis and quantifications of size diameters and zeta potentials. Error bars represent three independent experiments.

CS, HA-CS, and Alg-CS NPs were prepared using a previously optimized methodology^8^ and subsequently characterized in buffers and in serum in order to understand their interactions with biological molecules. All prepared NPs showed narrow size distributions in buffers. CS NPs, as expected, showed large and positive zeta potentials (Fig. 1A). For the surface coating process, similar preparative conditions (e.g. pH during coating and MWs of the coating agent), using different polyanions were applied to maximize the resemblance in the internal structures and morphologies of the resulted particles^8, 10^. This would minimize the differences in their interactions with serum proteins that stem from structural factors such as altering protein accessibility to chitosan cationic sites and altering the surface density and conformation freedom of the adsorbed polyanions. Upon coating with anionic HA, the positive surface charge of CS NPs was shielded and the size diameter increased significantly (from 170 nm to 270 nm), which is likely due to moderate agglomeration during the adsorption process (Fig. 1B and C). Adsorption of another type of polyanion (i.e. Alg) changed the CS NPs charge from cationic to anionic as well. However, Alg-CS NPs showed higher negative zeta potentials and ~three times larger size in diameters when compared to their HA coated counterparts. The latter finding is most likely due to the higher charge density of Alg in comparison to HA. Alg would exhibit a more rapid and more efficient adsorption onto the cationic CS NPs, which will lead to increase both attraction and agglomeration of partially coated particles during the adsorption process.

**Figure 2:**
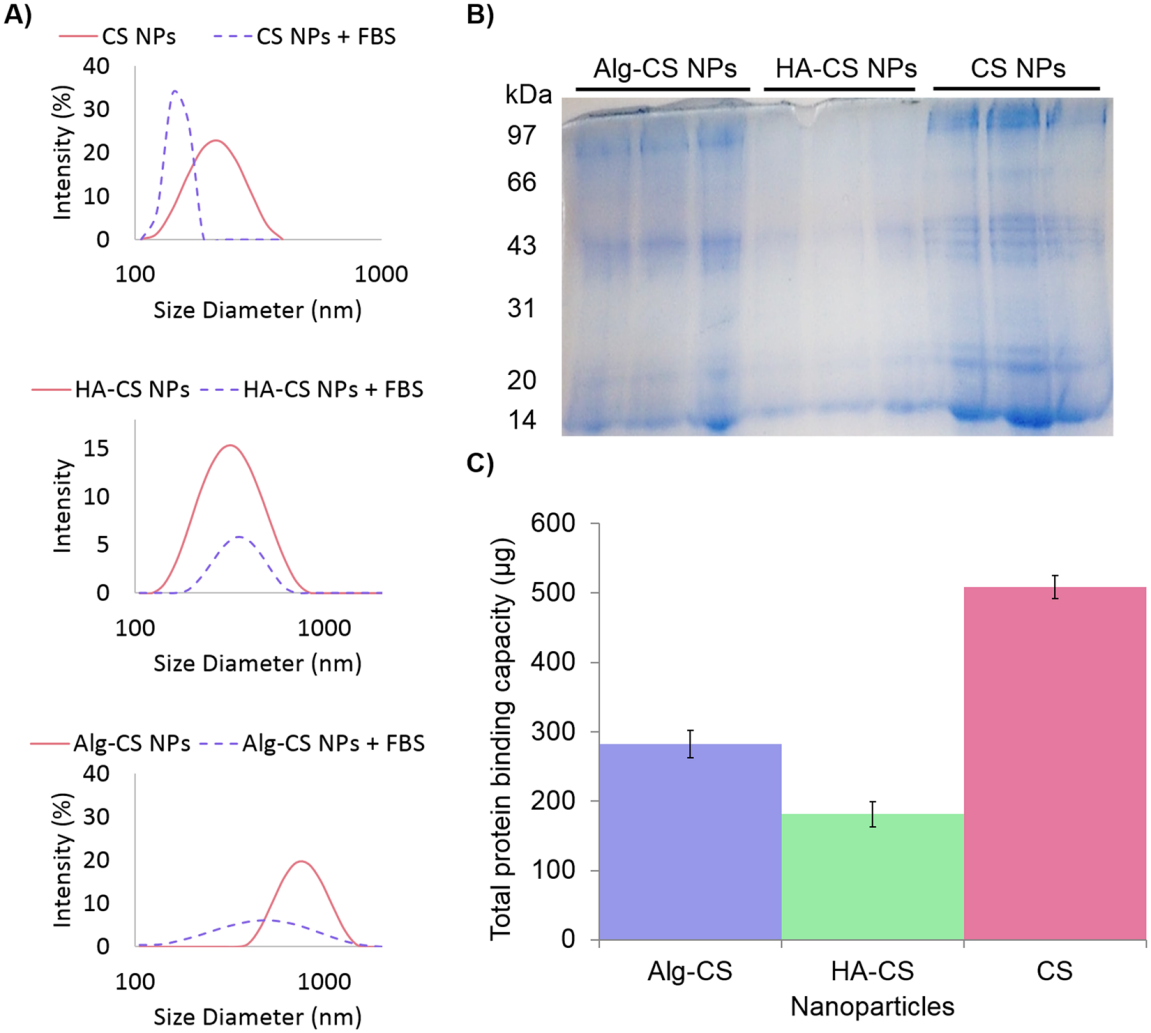
Pattern and loading of protein coronas. (**A**) Size distributions of CS, HA-CS, and Alg-CS NPs before and after incubation with serum. (**B**) SDS-PAGE gel electrophoresis of chitosan nanoparticles (CS NPs), hyaluronic acid-coated CS NPs (HA-CS NPs), and alginate-coated CS NPs (Alg-CS NPs). 12% gel was used, and 4 µg of protein marker was loaded. (**C**) Bar graph depicting the quantitative differences in the amount of corona proteins (µg/µL) that were adsorbed to each of the different NPs. Error bars represent three independent experiments.

The interaction of NPs with serum proteins was then analyzed following incubation with serum to form the PCs. Macroscopic agglomeration occurred instantly upon addition of serum to CS NPs. The size distribution recorded post serum incubation was significantly lower (Fig. 2A), which might be an indication of rapid agglomeration of nanoparticles followed by sedimentation. The net charge density of serum proteins is negative at physiological pH^4^. Particles with cationic surfaces often exhibit a rapid and intense adsorption of high affinity and highly abundant proteins upon exposure to biological environments, which results in a dense but less diverse PC^15^. Indeed, plain CS NPs showed the highest reactivity to serum proteins, which is highly attributed to their cationic nature (Fig. 2B,C). Comparatively, no significant changes in size distribution of HA-CS NPs were observed after incubation with serum. Moreover, HA coating significantly decreased protein adsorption compared to controls (CS and Alg-CS NPs), as the bands were quite pale and barely detectable (Fig. 2B). This is consistent with previous reports^10^ and could be attributed to the fact that HA-CS NPs exhibit moderate and negative charge densities relative to controls^16^. Alg-CS NPs considerably shrunk in size distribution after incubation with serum proteins (Fig. 2A). This effect could be attributed to the reduced nanoparticle swelling due to the increased external osmotic pressure, which would be more apparent in larger agglomerated structure^10^.

Mass spectrometric analysis showed differential adsorption of PCs that distinguishes each type of NPs based on their unique protein signatures. Supplementary Tables S2–4 list the proteomic profiles that have been identified from the coronas formed by CS, Alg-CS, and HA-CS NPs. Our stringent criterion to exclude random adsorption of proteins that were not present in all experimental triplicates (i.e. false proteins) showed that Alg-CS NPs were able to adsorb 25 stable proteins within the formed corona (Supplementary Figure S1). These numbers are higher than the ones adsorbed by either CS NPs or HA-CS NPs (18 and 16 proteins, respectively).

**Figure 3:**
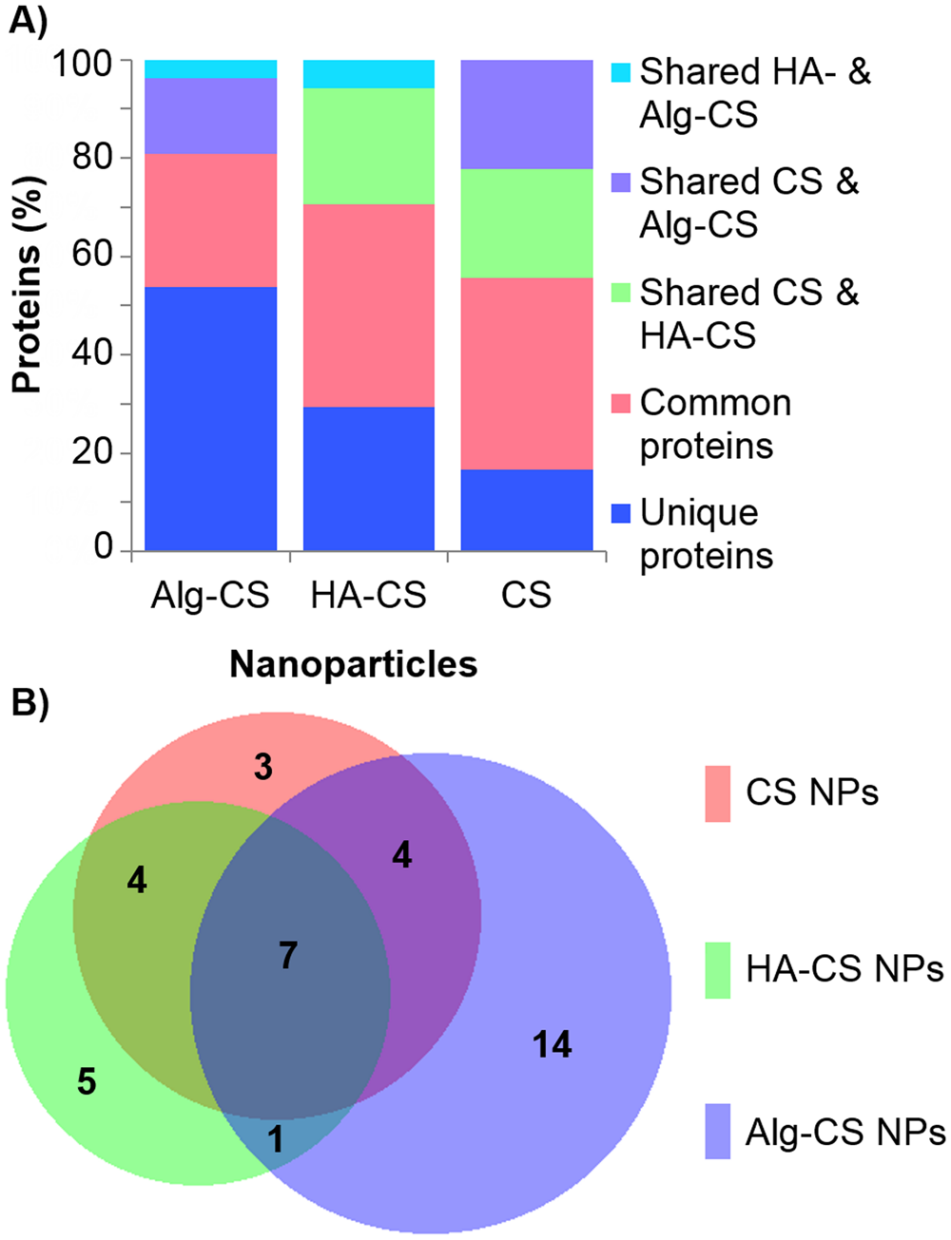
Mass spectrometric analysis. (**A**) Overlap comparison of protein coronas formed by chitosan nanoparticles (CS NPs), hyaluronic acid-coated CS NPs (HA-CS NPs), and alginate-coated CS NPs (Alg-CS NPs). (**B**) A Venn diagram depicting the differences and similarities in the number of the corona proteins identified between each nanoparticle.

Interestingly, the concentration of total protein loading within the PCs of each particle is not necessarily proportional to the absolute number of different protein identities. On the one hand, the concentration of total protein loading was 33% less for the HA-CS NPs relative to Alg-CS NPs (Fig. 2B), while HA-CS NPs adsorbed 36% less absolute number of different proteins compared to the ones adsorbed onto Alg-CS NPs (Fig. 3). On the other hand, however, CS NPs were roughly 40% higher than Alg-CS NPs in total protein loading, but were 28% less in adsorbing absolute number of different protein identities.

As for the unique protein signature for each system, the coronas of the CS, HA-CS, and Alg-CS NPs consisted of 17% (3 proteins), 31% (5 proteins), and 56% (14 proteins) distinctive proteins, respectively (Fig. 3). The diverse PC surrounding the Alg-CS NPs could be partially attributed to their considerably larger size, which offers a higher number of protein interaction sites per particle^17^, that is, a broader spectrum of proteins adsorbed. In addition to the differential adsorption of unique proteins, we investigated the degree of similarities between PCs formed around the different particles and identified seven common proteins adsorbed onto all three NPs (Fig. 3B and Supplementary Table S4). Four proteins were exclusively shared by the CS and Alg-CS NPs, and another four proteins were shared by CS and HA-CS NPs, while one protein was shared between HA-CS NPs and Alg-CS NPs. This is worth noting since HA-CS NPs and Alg-CS NPs are considered negatively charged NPs, but shared the lowest number of proteins as opposed to the positively charged CS NPs.

**Figure 4:**
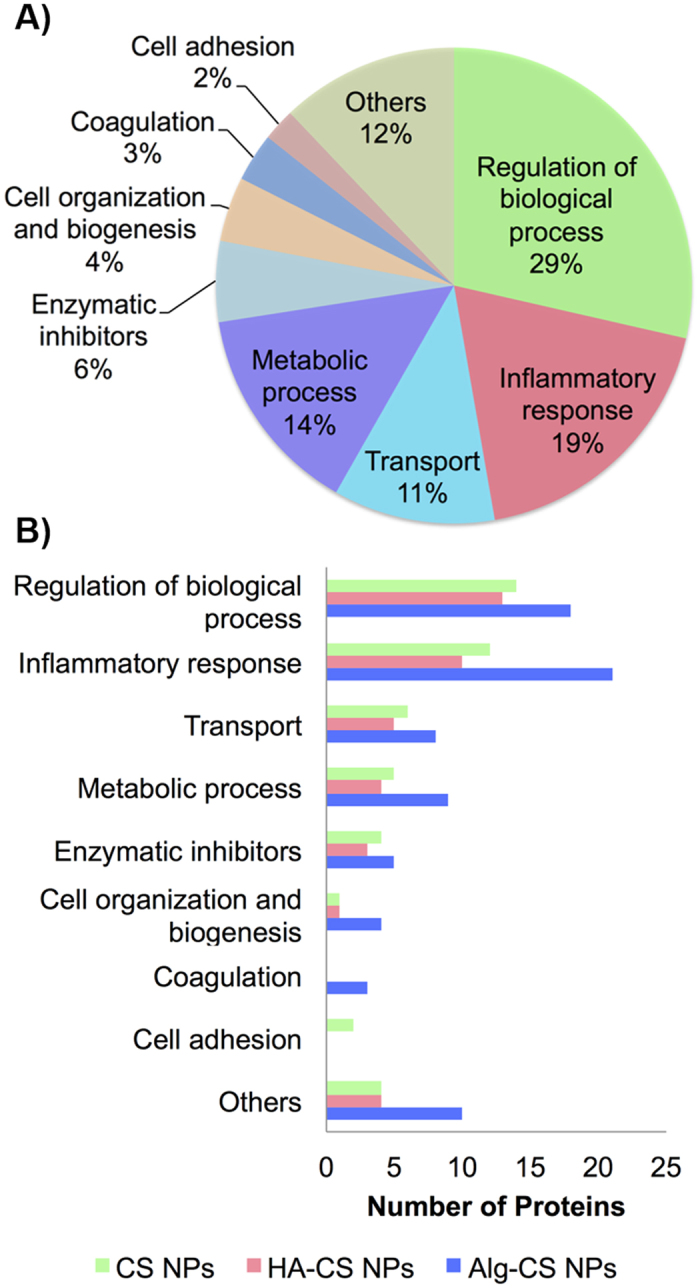
Gene Ontology terms analysis. (**A**) Pie chart depicting the percentage of involvement of the identified proteins and their molecular functions. (**B**) A bar graph comparing the different molecular functions of proteins adsorbed to NPs.

The molecular functions of the identified proteins was annotated by using GO enrichment terms in order to gain more insights into the nature of the formed PCs. Mapping of the identified proteins to GO terms generated 19 candidate pathways (Supplementary Tables S4 and S5, Supplementary Figure S2). Our results showed that 28% of the identified proteins were related to the regulation of biological processes, and 18% were involved in regulating the inflammatory response (Fig. 4A). However, some of the protein identities were also involved in important inflammation-related processes such as enzymatic inhibition, coagulation, and cell adhesion (8%, 3%, and 2%, respectively). To analyze this further, we looked at the GO terms that are related to the function of proteins adsorbed onto each NP (Fig. 4B). Such analysis revealed that HA coating of NPs reduced the deposition of proteins involved in inflammatory response, which includes immune process, defense response, immune process regulation, complement pathway, response to stimulus, and cell death. These results suggest that HA-CS NPs formed lower immunogenic PCs relative to both CS and Alg-CS NPs.

**Figure 5:**
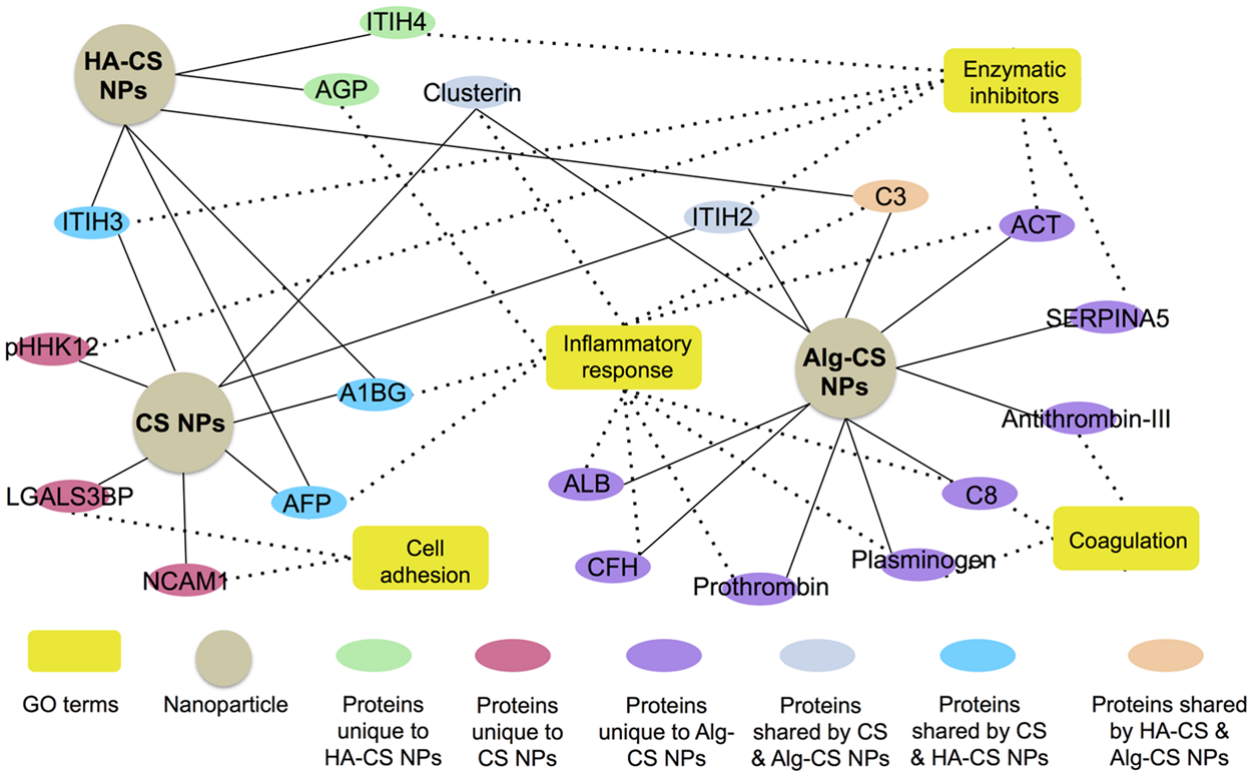
Inflammatory focused nanoparticle-protein-function network. A network map of chitosan nanoparticles (CS NPs), hyaluronic acid-coated CS NPs (HA-CS NPs), and alginate-coated CS NPs (Alg-CS NPs) was analyzed that adsorb nineteen serum proteins involved in four inflammatory-related molecular functions. Spherical nodes in the map represent NPs, oval nodes represent proteins, while yellow rectangular nodes represent molecular functions. Oval nodes were color coded to distinguish unique proteins from those shared by two different NPs. Solid lines represent proteins absorbed onto NPs, while the dotted lines represent the molecular functions related to the identified proteins.

**Figure 6:**
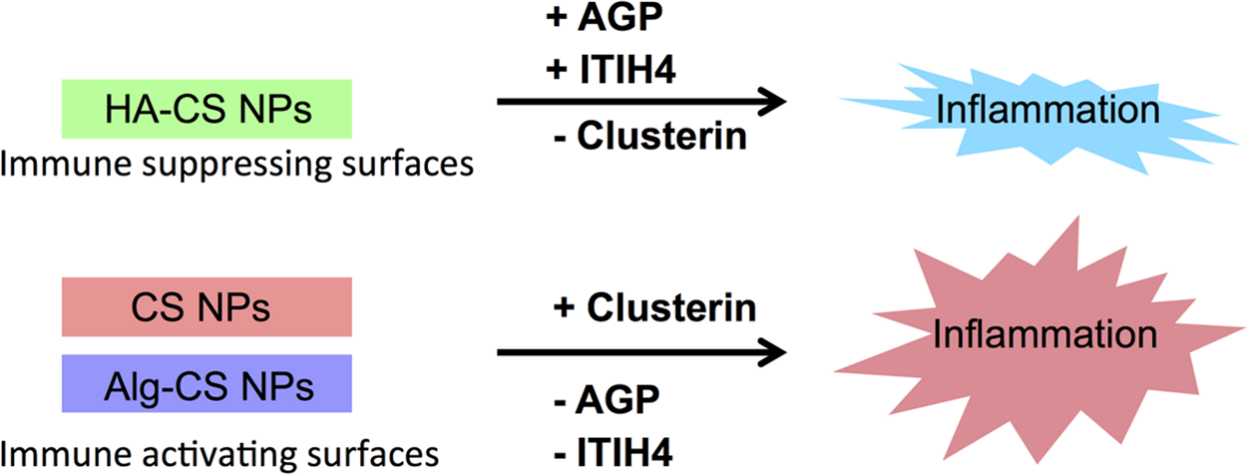
Proposed mechanism of creating immune modulating nanomaterials. Functionalizing of NPs with either anti-inflammatory proteins (AGP/ITIH4) or a proinflammatory protein (Clusterin) to produce immune suppressing/activating surfaces.

Next, we examined the identities of the unique protein signature of HA-CS NPs in order to further understand their potential utility as weakly immunogenic nanocarriers. We focused on GO terms related to proteins enriched for inflammatory response, enzymatic inhibition, coagulation, and cell adhesion. Such analysis identified alpha-1-acid glycoprotein (AGP) and inter-alpha-trypsin inhibitor heavy chain H4 (ITIH4) as uniquely adsorbed proteins onto HA-CS NPs, which were absent from the PCs of both CS and Alg-CS NPs (Fig. 5). AGP, known also as Orosomucoid (ORM1), is a well-established anti-inflammatory protein to protect against the lethal side effects of acute inflammation^18, 19^. Published reports suggest that AGP exhibits an unusually high degree of glycan branches, which could form glycosidic bonds with HA^20^. ITIH4 is also an anti-inflammatory protein, which is known to be upregulated during surgical trauma^21, 22^. Such unique adsorptions of ITIH4 with HA-CS NPs are attributed to the ester bond between carboxyl groups of the aspartates at their C termini and the C-6 hydroxyl groups of the internal N-acetylglucosamines of HA^23^. On the other hand, both controls (CS and Alg-CS NPs) adsorbed Clusterin, which was not identified within the PC of HA-CS NPs. Clusterin has been linked to elicit various host immune processes including immune regulation, cell adhesion, and active cell death^24, 25^. As a result, a potential mechanism for synthesizing weakly immunogenic NPs when functionalized with AGP and/or ITIH4 is proposed (Fig. 6). These findings are being validated by a current work investigating the potential use of such nano drug delivery systems as immune suppressing surfaces.

Although it is not possible to draw a full picture for the physiological fate of the investigated NP formulations, it should be considered that all three NPs have shown affinities to some essential components of the complement pathway such as complement component C3, which has a central role in complement pathway initiation. Alternative pathways could also be predicted as all three nanoparticles have adsorbed alpha-2-HS-glycoprotein (AHSG) that is known to promote endocytosis and could have a lymphocytic stimulating effect^26^. Correlating these observations along with comprehensive analysis of the total protein content within each PC with possible metabolic and immunological pathways will be the subject of future studies. Quantitative proteomics would also reveal important connections between NP surface properties and the amount of each protein adsorbed onto NPs. Understanding the effect of surface structure properties of NPs on protein adsorption would definitely assist the industry of nano drug delivery systems and allow the development of NPs to deliver therapeutics with high efficacy.

## Methods

### Materials

High purity chitosan (deacetylation degree over 60% mol, from white mushroom, protein content less than 1% and viscosimetric molecular weight 60–120 kDa), sodium triphosphate pentabasic (TPP), alginic acid sodium (medium viscosity, molecular weight ≈ 200 kDa), 1 M hydrochloric acid (HCl), and 1 M sodium hydroxide (NaOH) were obtained from Sigma-Aldrich (UK). Hyaluronic acid (HA; weight-average molecular weight ≈ 200 kDa) was purchased from Medipol SA (Switzerland). Glacial acetic acid and sodium acetate were purchased from VWR BDH Chemicals (UK). Regenerated cellulose (RC) dialysis membrane (MWCO 1000 kDa) was obtained from SpectraPor, Spectrum Laboratories Inc. (USA). Bovine serum (BS) was obtained from Life Technologies (USA).

### Preparation of nanoparticles

#### Plain chitosan nanoparticles (CS NPs)

A 0.07% wt. chitosan (CS) solution was prepared by dissolving chitosan in 4.6 mM HCl and kept stirring overnight at room temperature. The pH was then adjusted to 5 by adding appropriate volumes of 0.1 M NaOH. A 0.1% wt. TPP solution in deionized water was prepared and the pH of the solution was adjusted to 5 using 0.1 M HCl. All solutions were filtered through a 0.22 µm pore size filter. 0.214 mL of TPP solution was added to 2.786 mL of CS solution, where the final concentrations of TPP and CS are 0.0071 and 0.064% wt., respectively, resulting in a 1:9 mass ratio of TPP:CS. Then, under magnetic stirring and agitation, complexation to form the CS NPs was carried for 30 min at room temperature. The final complexation was undisturbed for an additional 24 h after a brief sonication. The dispersed CS NPs were then dialyzed against deionized water (MWCO 1000 kDa).

#### Coated chitosan nanoparticles

Prepared CS NPs were dispersed at a concentration of 0.025% wt. in a 0.1 M acetic acid/acetate buffer at pH 5. The dispersions were then slowly added under vigorous stirring for 30 min at 1200 rpm to an equal amount and an equal strength of acetate buffer, containing HA or Alg at a concentration of 1.5 mg/ml to form HA-CS or Alg-CS NPs, respectively. The formed NPs were then dialyzed against deionized water (MW cut-off 1000 kDa) following published reports^10^.

### Characterization of nanoparticles

#### Dynamic light scattering (DLS)

Hydrodynamic diameter (Z-average size), polydispersity index (PDI), and zeta potential measurements were always performed at room temperature using a Zetasizer Nano ZS instrument (Model ZEN3600, Malvern Instruments Ltd., UK) fitted out with a solid state HeNe laser (λ = 633 nm) at a scattering angle of 173°.

#### Protein adsorption and SDS-PAGE analysis

0.5 mL of a 0.5 mg/mL NPs dispersed in deionized water were incubated for 30 min with equal volumes of Bovine serum (BS) at 37 °C under mild shaking. DLS measurements were carried out to determine the size distributions of NPs before and after incubation with serum. Next, NPs incubated with serum were centrifuged (11,000 rpm, for 60 min), and the resulting pellet was washed three times with 1 mL deionized water to remove proteins that were not firmly adsorbed onto the surface of NPs. After the last wash, the NPs were suspended in 100 mL of 1% SDS and 100 mM Tris buffer (pH 7.5) for 1 h at room temperature to elute the bound proteins from the NP-protein corona. No solid residues were present even after centrifugation at 11,000 rpm at 41 °C for 60 min to precipitate the protein, 400 µL of cold acetone was added to the eluent and kept at −20 °C overnight. After centrifugation, the protein pellet was washed three times with 0.5 mL cold acetone. After drying the acetone from the tube, the protein pellet was dissolved in a working buffer (7 M Urea, 2 M Thiourea, 30 mM Tris–HCl, 4% CHAPS, pH 8.5). The protein concentrations were determined in triplicate for each sample using the 2 D quant kit following manufacturer procedure (GE Healthcare, Sweden). The gel electrophoresis was next carried out in triplicate on a 12% gradient SDS-PAGE gel (1 mm gel thickness, 120 volts for 1 h) using PowerPack HV (Bio-Rad Laboratories). After electrophoresis, the gels were fixed in 40% methanol and 10% acetic acid and the proteins were visualized by Coomassie® Brilliant Blue G (Sigma-Aldrich, USA). Following staining, the gel was washed in MilliQ water and stored at 4 °C until used.

#### Liquid chromatography and high-resolution mass spectrometry

Nine proteins bands from the gels described above were excised and then sliced into small pieces and de-stained by rinsing in 50% acetonitrile (ACN) containing 100 mM NH_4_HCO_3_. After that the gel pieces were dehydrated in 100% ACN and left to dry for 30 min at room temperature. The protein in the gels were reduced and alkylated with 10 Mm dithiothreitol in 100 mM NH_4_HCO_3_ at 56 °C for 30 min, and alkylated with 55 mM iodoacetamide in 100 mM NH_4_HCO_3_ at room temperature for 45 min in the dark. Dehydration of the gel pieces were performed twice in 50% ACN containing 25 mM NH_4_HCO_3_ for 30 min, and then once in 100% ACN for 5 min. After drying for 30 min at room temperature, the gel pieces were rehydrated in a modified sequencing grade trypsin from Promega (20 ng/*μ*L in 25 mM NH_4_HCO_3_) on ice for 30 min. Digestion was performed overnight at 37 °C after removing the excess solution. The peptides (protein fragments) were extracted twice with 50% ACN containing 0.1% formic acid for 30 min and then dried in a vacuum concentrator and dissolved in a solution of 0.1% formic acid (FA). The peptides were then separated using a Thermo Scientific Easy nLCTM1000 system equipped with a separation column (Thermo Scientific) (Acclaim PepMap RSLC C_18_, particle size 2 *μ*m, pore size 100 Å, 50 *μ*m inner diameter (ID) × 15 cm length) and a trapping column (Thermo Scientific) (EASY-Column, 2 cm length, 100 *µ*m ID, particle size 5 *µ*m, pore size 120 Å, C_18_-A1). The Thermo Scientific Easy nLCTM1000 system was coupled to a LTQ Orbitrap XL mass spectrometer (Thermo Scientific), using buffer A (0.1% FA in water) and buffer B (100% ACN, 0.1% FA). For in-line desalting and concentration, 2 *μ*L of digest were loaded onto the trap column and then washed for 5 min with 100% buffer A at 5 *μ*L/min flow rate. Peptides were eluted at 300 nL/min flow rate with the following 80 min gradient: 2% buffer B for 5 min, gradient to 60% buffer B over 60 min, gradient to 80% buffer B in 2 min, 80% buffer B for 5 min, gradient from 80% to 4% buffer B in 1 min, 2% buffer B for 7 min. Full scan mass spectra were acquired in the Orbitrap over 200–1800 m/z. The four most intense ions at a threshold above 1000 m/z were selected for collision-induced fragmentation in the linear ion trap at normalized collision energy of 35% after accumulation to a target value of 1000 m/z. Dynamic exclusion was enabled with a repeat count of 1 and an exclusion mass width by mass 1.50 below and above the precursor ion m/z. The same precursor was excluded for 30 sec.

#### Mass spectrometry data analysis

Raw data files were subjected to the Proteome Discoverer software version 1.4 (Thermo Scientific) to set up the workflow, files were then submitted to Sequest by the Proteome Discoverer Daemon (Thermo Scientific). Peak lists in the range from 350–5000 m/z were searched against the Bovine.fasta (http://www.uniprot.org/). Carbamidomethylation of cysteine was set as a fixed modification, and oxidation of methionine was set as a variable modification. Peptide tolerance was allowed to be ±15 ppm; fragment mass tolerance was allowed to be ±0.8 Da, with a maximum of 2 missed cleavages. The Percolator peptide confidence filter was set to “medium”. All Proteome Discoverer search parameters are provided in Supplementary Table S1 and all identified proteins are given in Supplementary Tables S2–4 For statistical analysis, a stringent criterion was applied to distinguish false protein identities from true ones within each formed protein coronas. True proteins need to be present in all experimental triplicates in order to be analyzed further. To gain further insight into the nature and characteristics of the identified proteins on each of the different nanoparticles, bioinformatics analysis using Gene-Ontology (GO) enrichment terms was carried out. The proteins identified from the nanoparticles through the 1D gel-Nano LC-Tandem Mass Spectrometry were then annotated based on their respective subcellular localization, molecular function, and biological processes using the Uniprot GO classification with minor modifications. The term “Inflammatory Response” is used to represent six GO terms combined (Immune process, defense response, immune process regulation, complement pathway, response to stimulus, and cell death). Supplementary Figure S2 was generated with after removing uncharacterized proteins. The inflammatory NP-protein-GO network map was generated based on four molecular functions (inflammatory response, coagulation, cell adhesion, and enzymatic inhibitors) after excluding the common proteins shared by all NPs in addition to excluding uncharacterized proteins.

## Electronic supplementary material

Supplementary Information
