# Supplementary material for: Hyaluronic Acid Coated Chitosan Nanoparticles Reduced the Immunogenicity of the Formed Protein Corona
Source: Sci Rep. 2017 Sep 5;7:10542. doi: 10.1038/s41598-017-10836-7 (PMC5585335; doi:10.1038/s41598-017-10836-7)
Supplement: Supplementary file 1 — Supplementary Information [file 41598_2017_10836_MOESM1_ESM.pdf]

Supplementary Information

Hyaluronic Acid Coated Chitosan Nanoparticles Reduced the Immunogenicity of the Formed Protein Corona

Abdulaziz Almalik[1], Hicham Benabdelkamel[2], Afshan Masood[2], Ibrahim O. Alanazi[3], Ibrahim Alradwan[1],Majed A. Majrashi[1], Assim A. Alfadda[2,4], Waleed M. Alghamdi[3], Haitham Alrabiah[5], Nicola Tirelli[6], and Ali H. Alhasan[1]*

Figures and Tables:

Supplementary Figure S1. Mass spectrometric analysis of stably adsorbed proteins vs. false ones.

Supplementary Figure S2. Nanoparticle-protein-function network.

Supplementary Table S1. Mass spectrometric list of all the identified proteins in the different nanoparticles.

Supplementary Table S2. Composition of the formed CS NPs-protein corona (in triplicates).

Supplementary Table S3. Composition of the formed HA-CS NPs-protein corona (in triplicates).

Supplementary Table S4. Composition of the formed Alg-CS NPs-protein corona (in triplicates).

Supplementary Table S5. Identities of adsorbed proteins onto CS, HA-CS, or Alg-CS NPs. Orange (common proteins). Green (shared between CS and HA-CS NPs). Blue

(shared between CS and Alg-CS NPs). No proteins were shared between HA-CS and Alg-CS NPs.

Supplementary Table S6. Gene Ontology analysis of the adsorbed proteins onto CS, HA-CS, or Alg-CS NPs. Orange (common proteins). Green (shared between CS and HA-CS NPs). Blue (shared between CS and Alg-CS NPs). No proteins were shared between HA-CS and Alg-CS NPs.

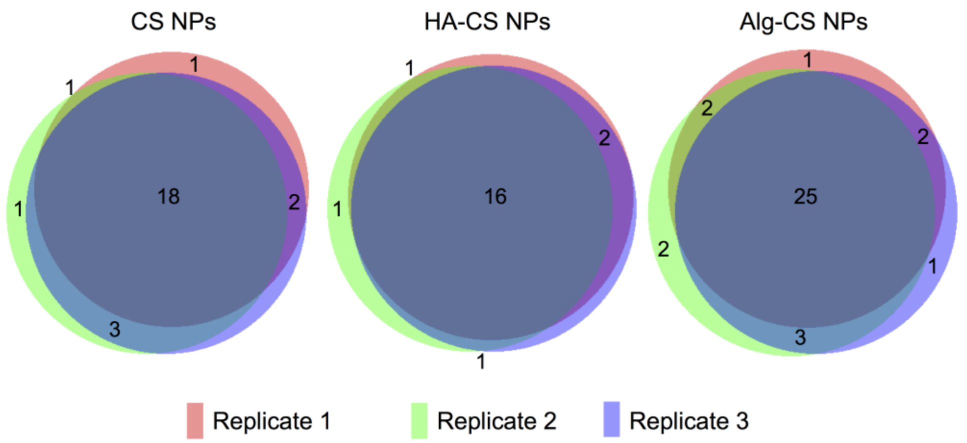

Supplementary Figure S1. Mass spectrometric analysis. Venn diagrams comparison of protein coronas formed in triplicates by either chitosan nanoparticles (CS NPs), hyaluronic acid-coated CS NPs (HA-CS NPs), or alginate-coated CS NPs (Alg-CS NPs) depicting the differences and similarities in the number of the corona proteins identified between each triplicate in order to distinguish stably adsorbed proteins from false ones.

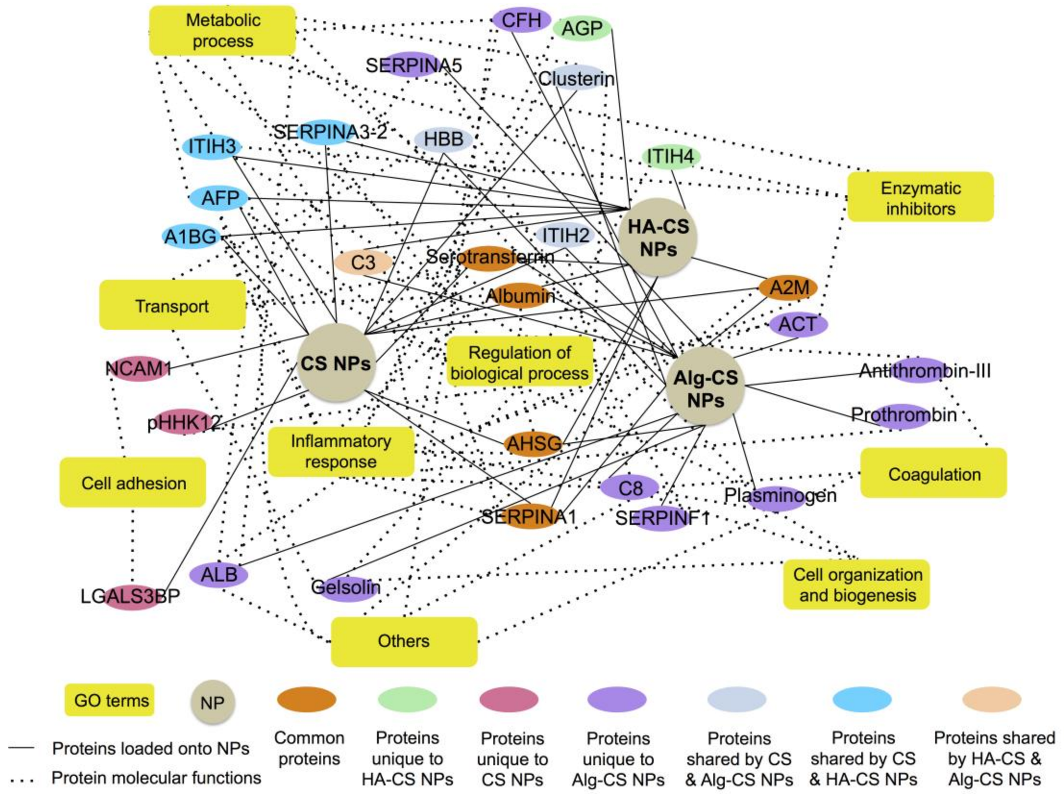

Supplementary Figure S2. Nanoparticle-protein-function network. A map network of chitosan nanoparticles (CS NPs), hyaluronic acid-coated CS NPs (HA-CS NPs), and alginate-coated CS NPs (Alg-CS NPs) was analyzed that formed protein coronas involved in various molecular functions. Spherical nodes in the map represent NPs, oval nodes represent proteins, while yellow rectangular nodes represent molecular functions. Oval nodes were color coded to distinguish unique proteins from both common proteins and shared proteins by two different NPs. Solid lines represent proteins absorbed onto NPs, while the dotted lines represent the molecular functions related to the identified proteins.

Supplementary Table S1: Composition of the formed protein corona by chitosan nanoparticles (CS NPs) in triplicates.

CS NPs-1		CS NPs-2		CS NPs-3	
**ID**	**Protein**	**ID**	**Protein**	**ID**	**Protein**
Q7SIH1	Alpha-2-macroglobulin	Q7SIH1	Alpha-2-macroglobulin	Q7SIH1	Alpha-2-macroglobulin
P02081	Hemoglobin fetal subunit beta	P02081	Hemoglobin fetal subunit beta	P02081	Hemoglobin fetal subunit beta
P34955	Alpha-1-antiproteinase	P34955	Alpha-1-antiproteinase	P34955	Alpha-1-antiproteinase
G3X6N3	Serotransferrin	G3X6N3	Serotransferrin	G3X6N3	Serotransferrin
P02769	Serum albumin	P02769	Serum albumin	P02769	Serum albumin
A5D7R6	ITIH2 protein	A5D7R6	ITIH2 protein	A5D7R6	ITIH2 protein
P12763	Alpha-2-HS-glycoprotein	P12763	Alpha-2-HS-glycoprotein	P12763	Alpha-2-HS-glycoprotein
A7E3W2	Galectin-3-binding protein	A7E3W2	Galectin-3-binding protein	A7E3W2	Galectin-3-binding protein
A2I7M9	Serpin A3-2	A2I7M9	Serpin A3-2	A2I7M9	Serpin A3-2
Q2KJF1	Alpha-1B-glycoprotein	Q2KJF1	Alpha-1B-glycoprotein	Q2KJF1	Alpha-1B-glycoprotein
Q3ZBS7	Uncharacterized	Q3ZBS7	Uncharacterized	Q3ZBS7	Uncharacterized
P56652	Inter-alpha-trypsin inhibitor heavy chain H3	P56652	Inter-alpha-trypsin inhibitor heavy chain H3	P56652	Inter-alpha-trypsin inhibitor heavy chain H3
Q27984	Alpha1-antichymotrypsin	Q27984	Alpha1-antichymotrypsin	Q27984	Alpha1-antichymotrypsin

	isoform pHHK12 (Fragment)		isoform pHHK12 (Fragment)		isoform pHHK12 (Fragment)
Q3SZ57	Alpha-fetoprotein	Q3SZ57	Alpha-fetoprotein	Q3SZ57	Alpha-fetoprotein
E1BMJ0	Uncharacterized	E1BMJ0	Uncharacterized	E1BMJ0	Uncharacterized
**Q2UVX4**	Complement C3			**Q2UVX4**	Complement C3
**E1BH06**	Uncharacterized	**E1BH06**	Uncharacterized		
**B0JYQ0**	ALB protein	**F1MI18**	Uncharacterized	**F1MI18**	Uncharacterized
P17697	Clusterin	P17697	Clusterin	P17697	Clusterin
**Q29S21**	Keratin, type II cytoskeletal 7	**E1B726**	Plasminogen	**Q29S21**	Keratin, type II cytoskeletal 7
F1N1W7	Neural cell adhesion molecule 1 (Fragment)	F1N1W7	Neural cell adhesion molecule 1 (Fragment)	F1N1W7	Neural cell adhesion molecule 1 (Fragment)
G3N0V2	Uncharacterized	G3N0V2	Uncharacterized	G3N0V2	Uncharacterized
		**E1B7N9**	Uncharacterized	**E1B7N9**	Uncharacterized
		**G5E593**	Uncharacterized	**G5E593**	Uncharacterized

Supplementary Table S2: Composition of the formed protein corona by hyaluronic acid-coated CS NPs (HA-CS NPs) in triplicates.

HA-CS NPs-1		HA-CS NPs-2		HA-CS NPs-3	
**ID**	**Protein**	**ID**	**Protein**	**ID**	**Protein**
G3N1Y3	Uncharacterized protein (Fragment)	G3N1Y3	Uncharacterized protein (Fragment)	G3N1Y3	Uncharacterized protein (Fragment)
P12763	Alpha-2-HS-glycoprotein	P12763	Alpha-2-HS-glycoprotein	P12763	Alpha-2-HS-glycoprotein
P34955	Alpha-1-antiproteinase	P34955	Alpha-1-antiproteinase	P34955	Alpha-1-antiproteinase
Q7SIH1	Alpha-2-macroglobulin	Q7SIH1	Alpha-2-macroglobulin	Q7SIH1	Alpha-2-macroglobulin
P02769	Serum albumin	P02769	Serum albumin	P02769	Serum albumin
G3X6N3	Serotransferrin	G3X6N3	Serotransferrin	G3X6N3	Serotransferrin
A2I7M9	Serpin A3-2	A2I7M9	Serpin A3-2	A2I7M9	Serpin A3-2
E1BMJ0	Uncharacterized	E1BMJ0	Uncharacterized	E1BMJ0	Uncharacterized
Q3SZ57	Alpha-fetoprotein	Q3SZ57	Alpha-fetoprotein	Q3SZ57	Alpha-fetoprotein
Q3ZBS7	Uncharacterized	Q3ZBS7	Uncharacterized	Q3ZBS7	Uncharacterized
Q3SZR3	Alpha-1-acid glycoprotein	Q3SZR3	Alpha-1-acid glycoprotein	Q3SZR3	Alpha-1-acid glycoprotein
		**A5D7R6**	ITIH2 protein	**A5D7R6**	ITIH2 protein
Q2KJF1	Alpha-1B-glycoprotein	Q2KJF1	Alpha-1B-glycoprotein	Q2KJF1	Alpha-1B-glycoprotein
G3X7A5	Complement C3	G3X7A5	Complement C3	G3X7A5	Complement C3
F1MVK1	Uncharacterized protein (Fragment)	F1MVK1	Uncharacterized protein (Fragment)	F1MVK1	Uncharacterized protein (Fragment)

**E1BH06**	Uncharacterized	**E1BMJO**	Uncharacterized	**E1BH06**	Uncharacterized
**Q29S21**	Keratin, type II cytoskeletal 7			**Q29S21**	Keratin, type II cytoskeletal 7
P56652	Inter-alpha-trypsin inhibitor heavy chain H3	P56652	Inter-alpha-trypsin inhibitor heavy chain H3	P56652	Inter-alpha-trypsin inhibitor heavy chain H3
F1MMD7	Inter-alpha-trypsin inhibitor heavy chain H4	F1MMD7	Inter-alpha-trypsin inhibitor heavy chain H4	F1MMD7	Inter-alpha-trypsin inhibitor heavy chain H4
**G3N0V2**	Uncharacterized	**G3N0V2**	Uncharacterized		

Supplementary Table S3: Composition of the formed protein corona by alginate-coated CS NPs (Alg-CS NPs) in triplicates.

Alg-CS NPs-1		Alg-CS NPs-2		Alg-CS NPs-3	
**ID**	**Protein**	**ID**	**Protein**	**ID**	**Protein**
P02081	Hemoglobin fetal subunit beta	P02081	Hemoglobin fetal subunit beta	P02081	Hemoglobin fetal subunit beta
Q9N2I2	Plasma serine protease inhibitor	Q9N2I2	Plasma serine protease inhibitor	Q9N2I2	Plasma serine protease inhibitor
P34955	Alpha-1-antiproteinase	P34955	Alpha-1-antiproteinase	P34955	Alpha-1-antiproteinase
B0JYQ0	ALB protein	B0JYQ0	ALB protein	B0JYQ0	ALB protein
P02769	Serum albumin	P02769	Serum albumin	P02769	Serum albumin
Q2UVX4	Complement C3	Q2UVX4	Complement C3	Q2UVX4	Complement C3
A5D7R6	ITIH2 protein	A5D7R6	ITIH2 protein	A5D7R6	ITIH2 protein
G3X6N3	Serotransferrin	G3X6N3	Serotransferrin	G3X6N3	Serotransferrin
E1BH06	Uncharacterized	E1BH06	Uncharacterized	E1BH06	Uncharacterized
E1BMJ0	Uncharacterized	E1BMJ0	Uncharacterized	E1BMJ0	Uncharacterized
Q3ZBS7	Uncharacterized	Q3ZBS7	Uncharacterized	Q3ZBS7	Uncharacterized
		**F1MVK1**	Uncharacterized	**F1MVK1**	Uncharacterized
P12763	Alpha-2-HS-glycoprotein	P12763	Alpha-2-HS-glycoprotein	P12763	Alpha-2-HS-glycoprotein
Q95121	Pigment epithelium-derived factor	Q95121	Pigment epithelium-derived factor	Q95121	Pigment epithelium-derived factor
F1MSZ6	Antithrombin-III	F1MSZ6	Antithrombin-III	F1MSZ6	Antithrombin-III

A6QM09	Putative uncharacterized protein	A6QM09	Putative uncharacterized protein	A6QM09	Putative uncharacterized protein
F1MLW8	Uncharacterized	F1MLW8	Uncharacterized	F1MLW8	Uncharacterized
		**G3N0V0**	Uncharacterized	**G3N0V0**	Uncharacterized
Q1JPD0	Complement component 8, alpha polypeptide (Fragment)	Q1JPD0	Complement component 8, alpha polypeptide (Fragment)	Q1JPD0	Complement component 8, alpha polypeptide (Fragment)
Q28921	Alpha 1-antichymotrypsin (Fragment)	Q28921	Alpha 1-antichymotrypsin (Fragment)	Q28921	Alpha 1-antichymotrypsin (Fragment)
P17697	Clusterin	P17697	Clusterin	P17697	Clusterin
P00735	Prothrombin	P00735	Prothrombin	P00735	Prothrombin
F1MJH1	Gelsolin	F1MJH1	Gelsolin	F1MJH1	Gelsolin
Q28085	Complement factor H	Q28085	Complement factor H	Q28085	Complement factor H
Q7SIH1	Alpha-2-macroglobulin	Q7SIH1	Alpha-2-macroglobulin	Q7SIH1	Alpha-2-macroglobulin
E1B726	Plasminogen	E1B726	Plasminogen	E1B726	Plasminogen
**Q29S21**	Keratin, type II cytoskeletal 7	**P2988**	VITRONECTIN	**Q29S21**	Keratin, type II cytoskeletal 7
G3N0V2	Uncharacterized	G3N0V2	Uncharacterized	G3N0V2	Uncharacterized
**P08226**	Apoloprotein E	**F1N4M7**	Uncharacterized	**F1N4M7**	Uncharacterized
**F1MSR8**	Collagen alpha-1(II) chain	**F1MSR8**	Collagen alpha-1(II) chain		

**F1MMD7**	Inter-alpha-trypsin inhibitor heavy chain H4	**F1MMD7**	Inter-alpha-trypsin inhibitor heavy chain H4	**P34928**	Apoliprotein C1
**E1B7N9**	Uncharacterized	**P0868**	IGHG1	**E1B7N9**	Uncharacterized

Supplementary Table S4: List of adsorbed proteins onto chitosan nanoparticles (CS NPs), hyaluronic acid-coated CS NPs (HA-CS NPs), and alginate-coated CS NPs (Alg-CS NPs).

CS NPs		HA-CS NPs		Alg-CS NPs	
**ID**	**Protein**	**ID**	**Protein**	**ID**	**Protein**
G3X6N3	Serotransferrin	G3X6N3	Serotransferrin	G3X6N3	Serotransferrin
E1BMJ0	Uncharacterized	E1BMJ0	Uncharacterized	E1BMJ0	Uncharacterized
P02769	Serum albumin	P02769	Serum albumin	P02769	Serum albumin
Q3ZBS7	Uncharacterized	Q3ZBS7	Uncharacterized	Q3ZBS7	Uncharacterized
P34955	Alpha-1-antiproteinase (SERPINA1)	P34955	Alpha-1-antiproteinase (SERPINA1)	P34955	Alpha-1-antiproteinase (SERPINA1)
Q7SIH1	Alpha-2-macroglobulin (A2M)	Q7SIH1	Alpha-2-macroglobulin (A2M)	Q7SIH1	Alpha-2-macroglobulin (A2M)
P12763	Alpha-2-HS-glycoprotein (AHSG)	P12763	Alpha-2-HS-glycoprotein (AHSG)	P12763	Alpha-2-HS-glycoprotein (AHSG)
A2I7M9	Serpin A3-2 (SERPINA3-2)	A2I7M9	Serpin A3-2 (SERPINA3-2)	A6QM09	Uncharacterized
P56652	Inter-alpha-trypsin inhibitor heavy chain H3 (ITIH3)	P56652	Inter-alpha-trypsin inhibitor heavy chain H3 (ITIH3)	B0JYQ0	ALB protein (ALB)
Q2KJF1	Alpha-1B-glycoprotein (A1BG)	Q2KJF1	Alpha-1B-glycoprotein (A1BG)	E1B726	Plasminogen

Q3SZ57	Alpha-fetoprotein (AFP)	Q3SZ57	Alpha-fetoprotein (AFP)	E1BH06	Uncharacterized
A5D7R6	Inter-alpha-trypsin inhibitor heavy chain H2 (ITIH2)	F1MMD7	Inter-alpha-trypsin inhibitor heavy chain H4 (ITIH4)	A5D7R6	Inter-alpha-trypsin inhibitor heavy chain H2 (ITIH2)
G3N0V2	Uncharacterized	G3N1Y3	Uncharacterized	G3N0V2	Uncharacterized
P02081	Hemoglobin fetal subunit beta (HBB)	Q3SZR3	Alpha-1-acid glycoprotein (AGP)	P02081	Hemoglobin fetal subunit beta (HBB)
P17697	Clusterin	F1MVK1	Uncharacterized	P17697	Clusterin
A7E3W2	Galectin-3-binding protein (LGALS3BP)	G3X7A5	Complement C3	Q2UVX4	Complement C3
Q27984	Alpha1-antichymotrypsin isoform pHHK12 (Fragment)			Q28921	Alpha 1-antichymotrypsin (ACT) (Fragment)
F1N1W7	Neural cell adhesion molecule 1 (NCAM1) (Fragment)			Q95121	Pigment epithelium-derived factor (SERPINF1)
				P00735	Prothrombin
				F1MJH1	Gelsolin
				Q28085	Complement factor H (CFH)
				Q1JPD0	Complement C8
				Q9N2I2	Plasma serine protease inhibitor (SERPINA5)
				F1MSZ6	Antithrombin-III

		F1MLW8 Uncharacterized

Supplementary Table S5: Gene Ontology (GO) analysis of the adsorbed proteins onto chitosan nanoparticles (CS NPs), hyaluronic acid-coated CS NPs (HA-CS NPs), and alginate-coated CS NPs (Alg-CS NPs).

**CS NPs**		**HA-CS NPs**		**Alg-CS NPs**	
**ID**	GO term	**ID**	GO term	**ID**	GO term
G3X6N3	Transport; cellular homeostasis	G3X6N3	Transport; cellular homeostasis	G3X6N3	Transport; cellular homeostasis
E1BMJ0	Regulation of biological process	E1BMJ0	Regulation of biological process	E1BMJ0	Regulation of biological process
P02769	Transport; cell communication; response to stimulus; regulation of biological process	P02769	Transport; cell communication; response to stimulus; regulation of biological process	P02769	Transport; cell communication; response to stimulus; regulation of metabolism
Q3ZBS7	Transport; response to stimulus; regulation of biological process; cell organization and biogenesis	Q3ZBS7	Transport; response to stimulus; regulation of biological process; cell organization and biogenesis	Q3ZBS7	Cell organization and biogenesis, transport; response to stimulus; regulation of biological process;
P34955	Complement pathway, Regulation of biological process	P34955	Regulation of biological process	P34955	Complement pathway, Regulation of biological process

Q7SIH1	Complement pathway, Regulation of biological process; cell differentiation	Q7SIH1	Complement pathway, Regulation of biological process; cell differentiation	Q7SIH1	Complement pathway, Regulation of biological process; cell differentiation
P12763	Immune system regulator, Response to stimulus, Regulation of biological process, defense response	P12763	Immune system regulator, Response to stimulus, Regulation of biological process, defense response	P12763	Immune process regulation, Response to stimulus, Regulation of biological process, defense response
A2I7M9	Regulation of biological process	A2I7M9	Regulation of biological process	A6QM09	Others
P56652	Regulation of biological process; metabolic process	P56652	Regulation of biological process; metabolic process	B0JYQ0	Transport; cell communication; response to stimulus; regulation of metabolism
Q2KJF1	Complement pathway regulator negative, 'Regulation of biological process,	Q2KJF1	Complement pathway regulator negative, 'Regulation of biological process,	E1B726	Regulation of biological process; 'metabolic process; coagulation, cell organization and biogenesis; cell differentiation; cellular

					homeostasis; cellular component movement
Q3SZ57	Response to stimulus, transport; reproduction; metabolic process; regulation of biological process;	Q3SZ57	Response to stimulus, transport; reproduction; metabolic process; regulation of biological process;	E1BH06	Defense response; response to stimulus; metabolic process; regulation of biological process
A5D7R6	Regulation of biological process; metabolic process	F1MMD7	Regulation of biological process; metabolic process	A5D7R6	Regulation of biological process; metabolic process
G3N0V2	Response to stimulus, defense response; metabolic process; regulation of biological process; transport	G3N1Y3	Transport	G3N0V2	Defense response; metabolic process; regulation of biological process; response to stimulus; transport
P02081	Transport	Q3SZR3	Regulation of immune system process	P02081	Transport
P17697	Response to stimulus, cell death; regulation of biological process; metabolic process,	F1MVK1	Regulation of biological process	P17697	Regulation of biological process; response to stimulus; metabolic process, ell death

F1N1W7	Cell Adhesion	G3X7A5	Complement pathway, Response to stimulus, regulation of biological process; metabolic process; defense response; response to stimulus	Q2UVX4	Complement pathway, Regulation of biological process; metabolic process; defense response; response to stimulus
A7E3W2	Cell Adhesion			Q1JPD0	Immune process regulation, regulation of biological process; response to stimulus; coagulation; cell organization and biogenesis; cellular homeostasis
Q27984	Regulation of biological process			Q95121	Regulation of biological process
				P00735	Immune Process, Response to stimulus
				F1MJH1	Transport; cell organization and biogenesis;

					regulation of biological process
				Q28085	Immune process regulation; metabolic process; regulation of biological process; response to stimulus
				Q28921	Regulation of biological process
				Q9N2I2	Regulation of biological process, 'Transport;
				F1MSZ6	Regulation of biological process, coagulation
				F1MLW8	Others
